# Molecular response of a patient with e19a2-positive chronic myeloid leukemia to flumatinib: a case report and literature review

**DOI:** 10.3389/fmed.2025.1515002

**Published:** 2025-03-17

**Authors:** Yaqing Feng, Hongjin Wang, Lidong Zhang, Jinying Gong, Xi Liu, Caiqin Mu, Jun Qiao, Haitao Meng, Yanfang Zhang

**Affiliations:** ^1^Department of Hematology, The Third People’s Hospital of Datong, Datong, Shanxi, China; ^2^Clinical Research Center, The Third People’s Hospital of Datong, Datong, Shanxi, China; ^3^State Key Laboratory of Experimental Hematology, National Clinical Research Center for Blood Diseases, Haihe Laboratory of Cell Ecosystem, Institute of Hematology and Blood Diseases Hospital, Chinese Academy of Medical Sciences and Peking Union Medical College, Tianjin, China; ^4^Tianjin Union Precision Medical Diagnostics Co. Ltd, Tianjin, China

**Keywords:** chronic myeloid leukemia, e19a2 transcript, second-generation tyrosine kinase inhibitor, flumatinib, molecular response

## Abstract

**Objective:**

Chronic myeloid leukemia (CML) is a malignancy driven by the *BCR::ABL1*fusion gene, with the e19a2 transcript being a rare variant, accounting for 0.4% of CML cases. Patients with the e19a2 transcript often show poor response to first-line treatment with imatinib, and no standard therapy has been established for this subtype.

**Methods:**

We report a case of a 28-year-old female with e19a2-positive CML. The patient exhibited poor response and tolerance to dasatinib. After 6 months, she achieved partial cytogenetic response (PCyR) but developed grade 3 pleural effusion. Following treatment discontinuation and prednisone therapy, the patient continued dasatinib (80 mg/d). At 12 months, the patient achieved complete cytogenetic response (CCyR), but *BCR::ABL1* levels remained suboptimal, with recurrent pleural effusion. The patient was then switched to flumatinib (600 mg/d), achieving major molecular response (MMR) at 6 months and deep complete molecular response (MR4.5) at 24 months, with good tolerance.

**Conclusion:**

Flumatinib demonstrated excellent deep molecular response and good tolerability in e19a2-positive CML patients, suggesting that it may be one of the preferred treatment options for such patients.

## Introduction

The BCR::ABL1 fusion gene is the primary molecular marker of chronic myeloid leukemia (CML) (1). The typical BCR::ABL1 transcripts are e13a2 and e14a2, which encode the p210 BCR::ABL1 protein. However, e19a2 is a rare variant that encodes the p230 BCR::ABL1 protein. The breakpoint cluster region (BCR) is located between exons 17 and 20 in the *μ* region, producing a fusion protein with a molecular weight of 230 kDa (2). Since Saglio et al. first reported the e19a2 variant in 1990, the number of related cases has gradually increased (3). Before the advent of tyrosine kinase inhibitors (TKIs), the treatment of CML mainly relied on allogeneic stem cell transplantation, chemotherapy, and interferon. However, chemotherapy and interferon treatment could only achieve limited hematologic responses, while allogeneic stem cell transplantation was limited by the availability of matched donors and treatment-related complications.

The introduction of the first-generation TKI, imatinib, in 2001 significantly improved the prognosis of CML patients. However, approximately 20% of patients develop resistance, leading to the emergence of second-generation TKIs (4). Although second-generation TKIs often provide deeper molecular responses (MR), they are also associated with adverse events such as pleural effusion, cytopenias, and vascular spasms or occlusive events, as well as pulmonary arterial hypertension (1). This case report discusses an e19a2-positive CML patient who showed poor response and tolerability to dasatinib but achieved a favorable molecular response following treatment with flumatinib.

## Case presentation

**Figure 1:**
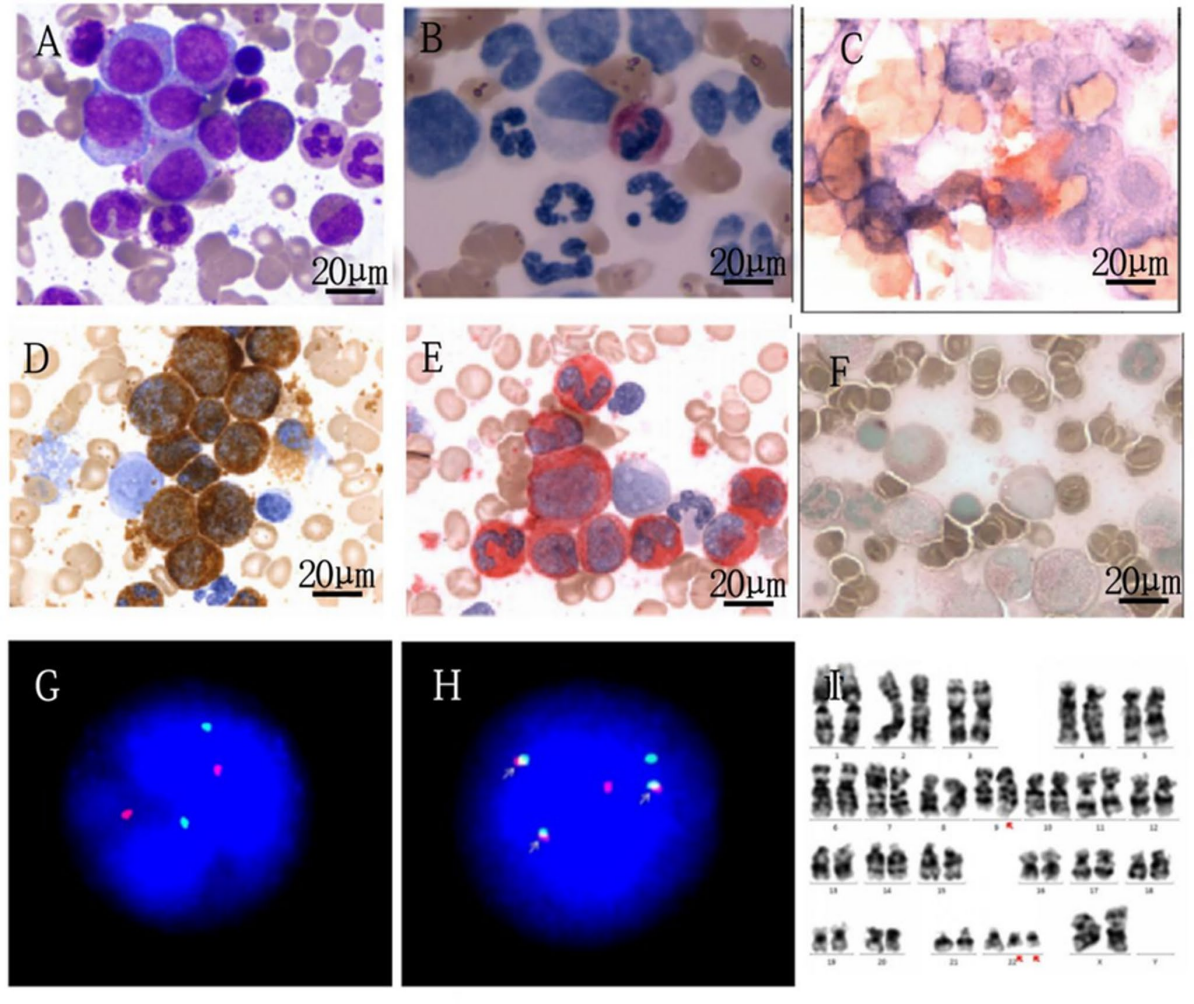
Laboratory examination of the patient. **(A)** Patient's bone marrow cell morphological analysis (1000x). **(B)** Patient's cytochemical staining (1000x). **(C)** CD41 (1000x). **(D)** Peroxidase staining (1000x). **(E)** Specific esterase staining (1000x). **(F)** Nonspecific esterase staining (1000x). **(G,H)** FISH analysis–normal control and patient’s image, respectively.

A 28-year-old female patient, during the Chinese New Year in 2020, experienced a gradual decrease in appetite while staying at home due to the COVID-19 pandemic, resulting in a weight loss of approximately 10 kg by July 2020. Subsequently, the patient developed worsening fatigue and nausea, accompanied by vomiting after eating, and eventually sought medical attention. On examination: anemia-like appearance, spleen tip 10 cm below the rib margin. No enlarged superficial lymph nodes were palpated, and no suspicious masses were detected. Laboratory tests showed: white blood cell count of 314.45 × 10^9^ g/L, with eosinophils at 1%, basophils at 11%, hemoglobin at 86 g/L, and platelet count at 524 × 10^9^ g/L. Peripheral blood analysis showed 5% blast cells. Ultrasound revealed splenomegaly (thickness 6.5 cm, length 22.8 cm), with Sokal risk score indicating intermediate risk (1.18), EUTOS score indicating high risk (117.0), and ELTS score indicating intermediate risk (1.76) (see Table 1). Ultrasound of the spleen revealed a thickness of 6.5 cm and length of 22.8 cm. Bone marrow aspiration showed hypercellularity with 7% blast cells (see Figure 1A). Neutrophil alkaline phosphatase (N-ALP) positivity rate was 2.00% (see Figure 1B). Fluorescence *in situ* hybridization (FISH) showed BCR::ABL1 fusion signals in 40% of cells (see Figures 1G,H). Cytogenetic analysis revealed a karyotype of 46, XX, *t*(9; 22)(q34; q11.2)/47, XX, *t*(9; 22), +der (22)t(9; 22) (see Figure 1I).

**Table 1:** The patient's laboratory test results and treatment response

**Date**	**WBC count(X10** ^**9**^ **/L)**	**Hemoglobin (g/L)**	**Platelet count(X10** ^**9**^ **/L)**	**Ultrasound of the spleen**		**BCR::ABL1 level of the e19a2 transcript**	**Cytogenetic response**	**Other characteristics**	**Intervention**	**Efficacy evaluation**	**Adverse reactions**
				**Thickness**	**Length**						
2020.07 (Baseline)	314	86	524	6.5 cm	22.8 cm	62.50%	100%	Sokal score:1.18 EUTOS score high-risk:117.0 ELTS score intermediate risk: 1.76	DS 100mg/d	_	_
2020.08 (Month 1)	18.58	91	176	_	_	_	_	_	_	_	_
2020.10(Month 3)	5.19	96	128	_	_	46.87%	100%	_	DS 100 mg/d	CHR	_
2021.01(Month 6)	5.13	110	105	4.5 cm	16 cm	3.84%	25%	_	DS, followed by pleural drainage, diuretics, and prednisone treatment. Subsequently, 80 mg/d.	PCyR	Grade 3 pleural effusion.
2021.07(Month 12)	3.63	93	82	3.8 cm	11 cm	0.17%	0%	_	Discontinued DS, switched to FM, and simultaneously administered iron supplements.	CCyR	Second occurrence of grade 3 pleural effusion, cytopenia, rash, and iron-deficiency anemia (possibly caused by excessive menstrual bleeding).
2022.01(Month 18)	6.75	112	172	_	_	0.1%	_	_	_	MMR	In the early stage of flumatinib treatment, mild gastrointestinal discomfort occurred and resolved quickly.
2022.07(Month 24)	9.17	112	195	_	_	0.04%	0%	_	FM	_	_
2023.01(Month 30)	10.02	126	207	_	_	0.01%	_	_	_	MR 4.0	_
2023.07(Month 36)	6.06	100	182	_	_	0%	0%	_	FM	MR 4.5	_

ABL1, Abelson murine leukemia viral oncogene homolog 1; BCR, break point cluster; CCyR, complete cytogenetic response; CHR, complete hematological response; DS, dasatinib; EUTOS, European Treatment and Outcome Study; ELTS, EUTOS long‐term survival; FM, flumatinib; MR, molecular responses; MMR, major molecular responses; PCyR, partial cytogenetic response; WBC, white blood cells.

Using a dual color and dual fusion BCR-ABL1 probe, the BCR (22q11) gene is marked with green, the ABL1 (9q34) gene is marked with red, and the *BCR::ABL1* fusion gene is represented by a yellow or red, green superimposed signal. The negative signal is a 2 red and 2 green signal modes. The ISCN result of this patient is nuc ish (ABL1, BCR) × 3 (ABL1 con BCR × 2) [221/500]/(ABL1, BCR) × 4 (ABL1 con BCR × 3) [2000/500], indicating a positive detection rate of 84.2% for the BCR-ABL1 fusion gene. Among them, 40% of positive cell fusion signals increased with the addition of a fusion copy. *ABL1 (9q34),* Abelson murine leukemia viral oncogene homolog 1 is located on chromosome; *9q34BCR (22q11)*, breakpoint cluster region gene is located on chromosome 22q11; FISH, fluorescence *in situ* hybridization.

**I** Chromosome Analysis Karyotype Description 46,XX,t(9;22)(q34;q11.2) (5)/47,XX,t(9;22),+der(22)t(9;22) (6).

**Figure 2:**
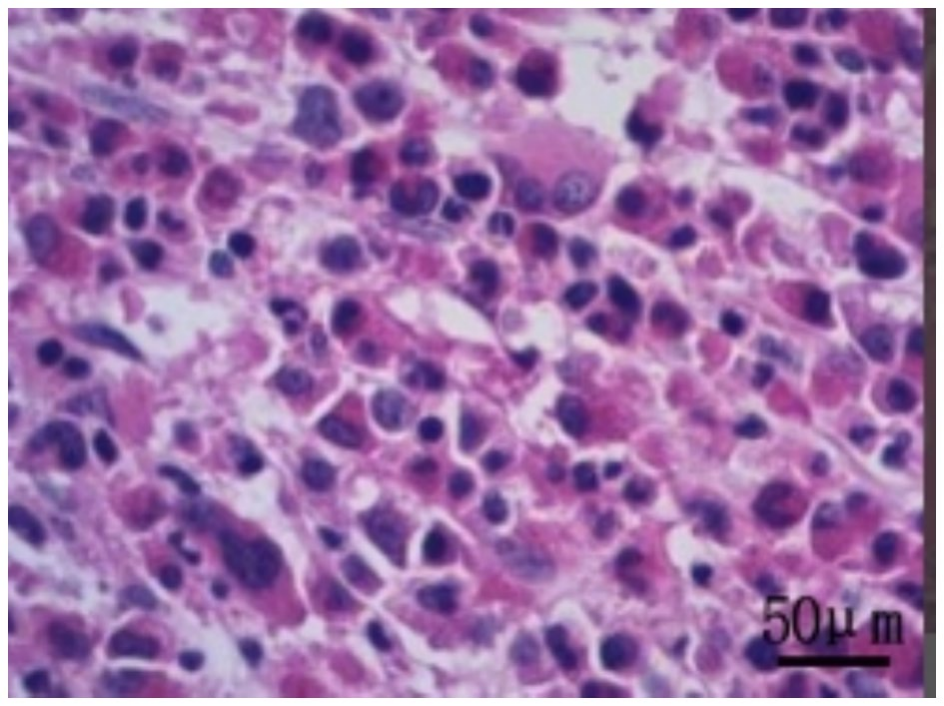
Bone marrow biopsy pathology of the patient (400X).

Bone marrow biopsy indicated active marrow proliferation with fibrosis, and reticulin staining was graded as MF-2 (see Figure 2). The BCR/ABL P210 fusion gene was negative, the BCR/ABL P190 fusion gene was negative, and the BCR/ABL P230 fusion gene was positive, confirming the diagnosis of e19a2-positive CML.

In July 2020, the patient began treatment with dasatinib at a dose of 100 mg/d. After 3 months of treatment, the patient achieved complete hematological response (CHR), but no cytogenetic response (CyR) was observed, with BCR::ABL1 > 10%. After 6 months, the patient achieved partial cytogenetic response (PCyR) with BCR::ABL1 < 10%, but grade 3 pleural effusion developed. The spleen thickness reduced to 4.5 cm and the length reduced to 16 cm. The tuberculin test was negative, and no acid-fast bacilli or fungi were detected. Liquid-based cytology showed proliferation of mesothelial cells and lymphocytes, but no malignant changes were observed. The pleural effusion was attributed to a side effect of dasatinib. The patient discontinued dasatinib treatment and was given diuretics and prednisone (40 mg/d). According to the 2020 European LeukemiaNet (ELN) guidelines, the treatment evaluation was in the warning zone, and a change in therapy was recommended. However, the patient chose to continue dasatinib treatment with a dose reduction to 80 mg/d. After 12 months, the patient achieved complete cytogenetic response (CCyR) with BCR::ABL1 reduced to 0.17%, but did not reach the optimal response. The spleen thickness further reduced to 3.8 cm and the length reduced to 11 cm. The patient developed grade 3 pleural effusion again, along with skin itching, cytopenia, and iron-deficiency anemia (possibly due to excessive menstrual bleeding). She resumed prednisone therapy, underwent pleural drainage, and received iron supplementation. The pleural effusion resolved after discontinuing dasatinib.

In July 2021, the patient switched to treatment with flumatinib at a dose of 600 mg/d. After 6 months, the patient achieved major molecular response (MMR), with BCR::ABL1 at 0.1%. After 18 months, the patient reached MR4.0, and at 24 months, BCR::ABL1 was undetectable, indicating achievement of MR4.5. During the early phase of flumatinib treatment, the patient experienced mild gastrointestinal side effects, which resolved quickly, and she tolerated the treatment well without recurrence of pleural effusion. The molecular response remained stable. At the same time, she resumed her daily activities, and her quality of life significantly improved.

## Patient’s perspective

The patient expressed satisfaction with the treatment outcome.

## Discussion

CML is a malignancy caused by clonal mutations of hematopoietic stem cells, characterized by the BCR::ABL1 fusion gene. CML is usually detected during physical exams or blood tests, and the presence of Ph chromosome abnormalities is confirmed by routine cytogenetic analysis, fluorescence *in situ* hybridization (FISH), or molecular studies (1). The typical BCR::ABL1 transcripts are e13a2 and e14a2, which encode the p210 fusion protein. Rare transcripts such as e19a2, e14a3, e1a2, and e13a3 account for 0.4, 0.3, 0.9, and 0.1% of CML cases, respectively. Other rare transcripts, including e1a3, e6a2, and e2a2, have also been reported (7). The e19a2 transcript encodes the p230 fusion protein, which differs clinically from the more common p210 isoform. Initially, the e19a2 subtype was often observed in neutrophilic CML, presenting with a benign clinical course. However, it has later been found predominantly in typical CML patients, some of whom exhibit more aggressive clinical manifestations. Patients with the p210 transcript tend to reach treatment goals more rapidly and have a lower treatment failure rate. In contrast, patients with the p230 transcript generally take longer to respond and have a higher risk of treatment failure. However, these patients show better responses to second-generation TKIs (2GTKIs) (2).

In the early stages, CML patients with the e19a2 transcript were treated with interferon and hydroxyurea, but these treatments only induced hematological responses, and most patients died due to disease progression. These patients also showed poor responses to the first-generation TKI imatinib, with most failing to achieve or maintain deep MR, leading to continued disease progression (2). Studies comparing patients with the p230 transcript to those with the p210 transcript revealed significant differences in treatment outcomes. The 1-year CCyR rate for p230 was 44.4%, while for p210 it was 87.9%; the 1-year MMR rate for p230 was 48.6%, compared to 6.3% for p210; and the 2-year event-free survival (EFS) rate for p230 was 94.4%, compared to 69.1% for p210 (8). These findings are consistent with another study’s results (5).

Due to the lower efficacy of imatinib in achieving MMR, 2GTKIs, such as nilotinib and dasatinib, have become the preferred first-line treatment options. Currently, the guidelines recommend the use of imatinib, dasatinib, bosutinib, and nilotinib as first-line TKIs for CML (1). Although the number of reported cases is limited, some studies show that second-generation TKIs can induce deeper molecular responses more quickly. In one study, 10 patients receiving nilotinib (300 mg daily, divided into two doses) as first-line treatment showed good therapeutic effects: one patient achieved MR4.5 within 3 months and maintained it for 31 months, while the other patients achieved MMR within 2 to 6 months. Similarly, two patients treated with dasatinib (100 mg daily) reached MMR within 6 months, with the duration of MMR ranging from 3 months to 43 months (9, 10).

**Figure 3:**
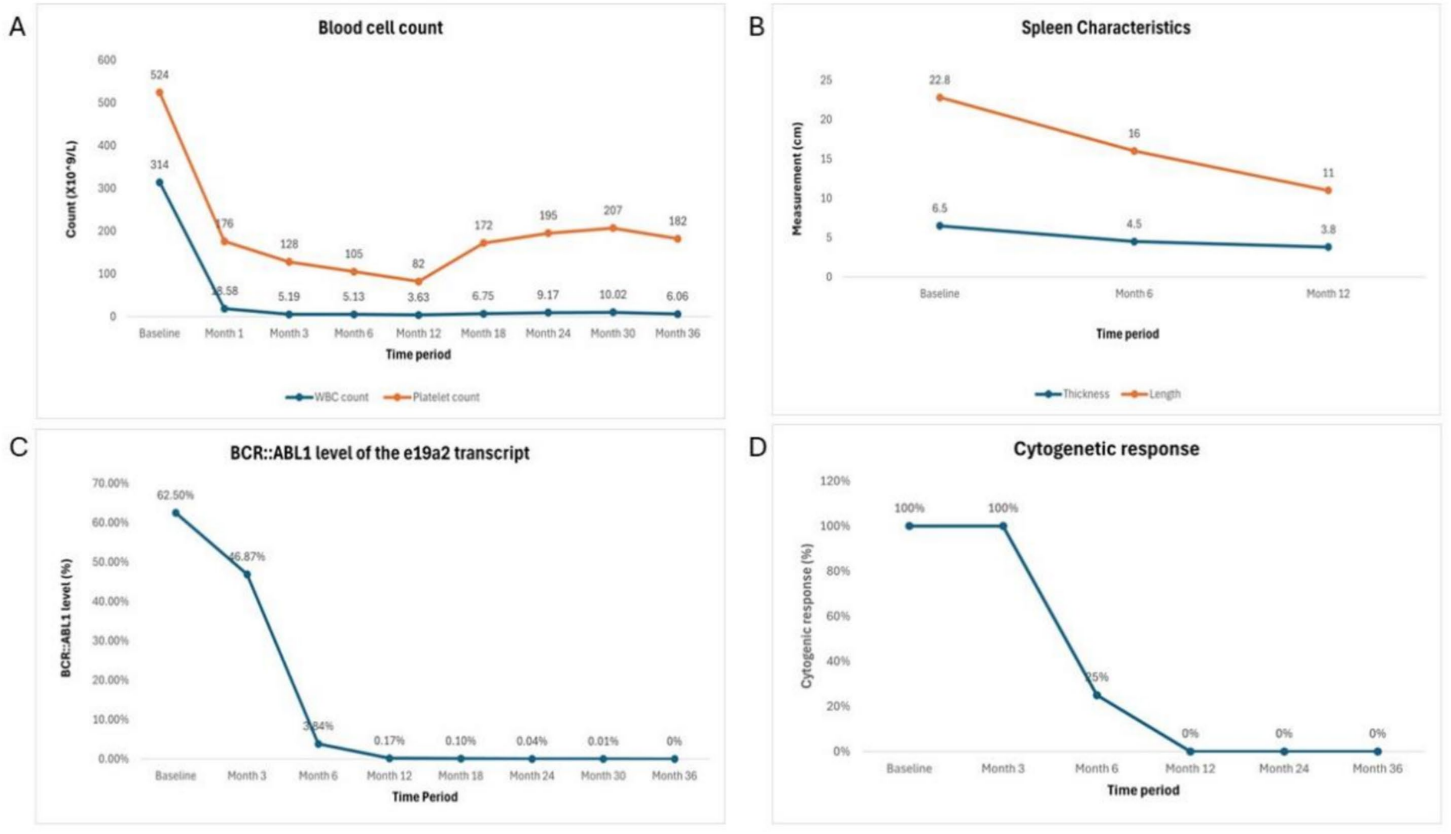
Laboratory test results and treatment response over treatment period. **(A)** Blood cell counts. **(B)** Spleen characteristics. **(C)** BCR::ABL1 level of e19a2 transcript. **(D)** Cytogenic response.

Our patient initially received dasatinib treatment and achieved CCyR at 12 months with BCR::ABL1 < 1%. However, due to suboptimal response, recurrent pleural effusion, rash, and poor tolerance, the treatment was switched to flumatinib. After 6 months of flumatinib treatment, the patient achieved MMR and reached MR4.5 at 24 months, with only mild side effects. This response is consistent with the ELN guidelines for CML treatment (11).The patient’s laboratory test results and treatment response (see Table 1 and Figure 3).

Due to its trifluoromethyl and imidazole ring structure, nilotinib demonstrates better lipophilicity and ATP-binding affinity, but its long-term use is associated with cardiovascular risks (12). Dasatinib, as a dual inhibitor of ABL and SRC kinases, has a lower specificity for the shape and charge of the binding sites, which makes it more likely to induce pleural effusion with lymphocytic infiltration. These immune-mediated effects are thought to be caused by SRC inhibition (13). The advent of the third-generation TKI, ponatinib, provides hope for overcoming the T315I mutation, but the second-phase PACE trial reported a cumulative incidence of arterial occlusive events of 31% in chronic-phase CML patients (6). Therefore, although ponatinib remains effective, its use requires careful risk assessment.

Flumatinib, optimized from imatinib, binds more strongly to the hydrophobic pocket of the tyrosine kinase domain, enhancing its stability and efficacy (14). *In vitro* studies have shown that flumatinib exhibits excellent inhibitory activity against BCR::ABL1 kinase point mutations (15). In a phase III clinical trial (FESTnd) evaluating newly diagnosed CML patients, flumatinib was mainly associated with mild adverse events (AEs) such as diarrhea, which typically lasted only 1–2 days. The incidence of grade 3 or higher adverse events was low, including grade 3 rash, diarrhea, and thrombocytopenia (all <1.5%). Rare cardiovascular events, such as atrial fibrillation or stroke (in individual cases) (16, 17), were also reported. In our case, the patient responded rapidly and completely to flumatinib, with no severe side effects. Compared to other second-generation TKIs, flumatinib provides excellent efficacy, overall good tolerability, and fewer serious adverse events (16, 17), making it a feasible treatment option for patients with underlying cardiovascular disease or poor physical condition. A summary of related literature is provided in Table 2.

**Table 2:** Summary of the included studies.

S. no	Authors and year	Title	Country	Total study population	Patient characteristics	Intervention	Main findings	Study conclusions
1	Yunfan Yang et al. (2024)	Safety and efficacy of flumatinib as later-line therapy in patients with chronic myeloid leukemia	China	336	Patients with CP- CML previously treated with TKI	Flumatinib 600 mg once daily	CHR, CCyR, MMR, and MR4/DMR was observed in 100%, 98.9%, 98.6%, and 92.9% patients, respectively. Tolerable AEs were observed	Flumatinib was effective and safe in patients resistant to other TKIs.
2	Xiaoshuai Zhang et al. (2024)	Comparison of the Efficacy Among Nilotinib, Dasatinib, Flumatinib and Imatinib in Newly Diagnosed Chronic-Phase Chronic Myeloid Leukemia Patients: A Real-World Multi-Center Retrospective Study	China	2,496	Patients with CP- CML receiving initial 2G-TKI	Nilotinib (*n* = 512), dasatinib (*n* = 134), flumatinib (*n* = 411) or imatinib (*n* = 1,439)	Patients receiving nilotinib, dasatinib or flumatinib therapy had comparable cytogenetic and molecular responses, FFS, PFS, and OS. Higher incidences of cytogenetic, higher molecular responses and FFS in patients receiving nilotinib, dasatinib or flumatinib than those receiving imatinib.	Efficacy (PFS and OS) among the 4 TKIs were comparable.
3	Li Zhang et al. (2021)	Flumatinib versus Imatinib for Newly Diagnosed Chronic Phase Chronic Myeloid Leukemia: A Phase III, Randomized, Open-label, Multi-center FESTnd Study	China	394	Patients aged 18–75 years, ECOG performance of 0–2, and Ph + CP-CML within 6 months of diagnosis	1:1 to flumatinib 600 mg once daily (*n* = 196) or imatinib 400 mg once daily (*n* = 198)	The 6- and 12-month MMR was significantly higher with flumatinib vs. imatinib (33.7% vs. 18.3%; 52.6% vs. 39.6%). AEs (edema, pain in extremities, rash, neutropenia, anemia, and hypophosphatemia) were more frequent in imatinib group; diarrhea and ALT elevation were more frequent in flumatinib group.	Patients treated with flumatinib achieved significantly higher response rates, along with faster and deeper response.
4	Ya-Qin Jiang et al. (2021)	Clinical Effect of Tyrosine Kinase Inhibitors in the Treatment of P230 Chronic Myeloid Leukemia	China	11	Patients with e19a2 transcript (P230) CML-CP	Imatinib 400 mg, qd (*n* = 4), nilotinib 300 mg, bid (*n* = 5), dasatinib 100 mg, qd (*n* = 2)	MMR was obtained within 1 year in patients treated with imatinib 400 mg, qd 6 months with nilotinib 300 mg, bid dasatinib 100 mg, qd	For patients with imatinib resistance, deeper molecular response, and MMR in a short time was achieved with 2G-TKIs
5	Mengxing Xue et al. (2019)	Clinical characteristics and prognostic significance of chronic myeloid leukemia with rare BCR-ABL1 transcripts	China	40	Patients with CML having rare BCR-ABL1 transcripts	Imatinib, nilotinib, dasatinib	Patients with e1a2 transcript had lower response rates after 3 months (*p* = 0.001) and a lower 1-year CCyR rate (19.0 vs. 79.9%,) than patients with the typical transcript. Patients with e19a2, e13a3/e14a3 transcript had similar response rates after 3 months and similar 1-year CCyR rates (80.0 vs. 79.9%, *p* = 0.820, and 66.7 vs. 79.9%, *p* = 0.571)	Patients with e19a2 transcript had a high rate of early optimal response to TKIs.
6	Jorge E. Cortes et al (2018)	Ponatinib efficacy and safety in Philadelphia chromosome–positive leukemia: final 5-year results of the phase 2 PACE trial	Republic of Korea, UK, Australia, Sweden, Spain, Netherlands, Italy, Germany, France, Singapore, Belgium	267	Patients with CP- CML resistant or intolerant to dasatinib or nilotinib, or who had the BCR-ABL1	Starting dose of 45 mg ponatinib once daily, and dose reductions to 30 mg or 15 mg once daily	60%, 40%, and 24% achieved MCyR, MMR, and 4.5-log molecular response. The probability of maintaining MCyR for 5 years: 82%. Estimated 5-year OS: 73%. Common treatment-emergent AEs were rash (47%), abdominal pain (46%), and thrombocytopenia (46%).	Ponatinib was safe and effective, irrespective of dose reductions, in patients with heavily pretreated CP-CML
7	Ya-Zhen Qin et al. (2018)	Prevalence and outcomes of uncommon BCR-ABL1 fusion transcripts in patients with chronic myeloid leukemia: data from a single center	China	4750	Patients with CML and uncommon BCR-ABL1 transcripts	Imatinib, nilotinib or dasatinib	Patients with the e19a2 transcript had low probabilities of 2-year EFS (*P* = 0.0004) and PFS (*P* = 0.0067)	Patients with the e19a2 or e1a2 transcript had poor treatment outcomes.
8	Sudha Sazawal et al. (2017)	Chronic myeloid leukemia with a rare fusion transcript, e19a2 BCR-ABL1: A report of three cases from India	India	03	Patients with p230 fusion protein and diagnosed with CML-CP.	Imatinib 400 mg per day	All patients achieved MMR at 12 months	Imatinib demonstrated significant efficacy in patients with e19a2 fusion transcript
9	Marianna Greco et al. (2013)	Early Complete Molecular Response to First-Line Nilotinib in Two Patients with Chronic Myeloid Leukemia Carrying the p230 Transcript	Italy	02	Patients with p230 transcript, diagnosed with CML-CP, who achieved fast and deep CMR with nilotinib	Nilotinib 600 mg per day	First patient achieved CCyR and CMR at 3 months. Second patient achieved CCyR and CMR in 6 and 8 months, respectively.	Nilotinib is recommended as frontline agent for the treatment of CML with e19a2 fusion transcript
10	Gaku Oshikawa et al. (2010)	Clonal evolution with double Ph followed by tetraploidy in imatinib-treated chronic myeloid leukemia with e19a2 transcript in transformation	Japan	01	Patient with e19a2 transcript CML	Imatinib	Leukocytosis recurred with 8.2% myeloblasts in the bone marrow two years after starting therapy. Duplication of Ph and tetraploidy occurred.	CML progressed in 2 years despite imatinib therapy
11	Mondal et al. (2006)	e19a2 BCR–ABL fusion transcript in typical chronic myeloid leukemia: a report of two cases	India	02	Patient with e19a2 transcript CML	The first patient was treated with hydroxyurea and progressed to the accelerated phase, followed by imatinib therapy. The second patient was treated with hydroxyurea	one of them developed accelerated phase CML and died 8 years after diagnosis and the other is at the chronic phase.	The disease progression of the e19a2 fusion transcript type may resemble that of typical CML
12	Ayda Bennour et al. (2010)	E355G mutation appearing in a patient with e19a2 chronic myeloid leukemia resistant to imatinib	Tunisia	01	CML patients with the e19a2 transcript, accompanied by the E355G mutation	Initially treated with hydroxyurea, followed by adjustment to imatinib (initial dose of 400 mg, later increased to 800 mg), and subsequently adjusted to nilotinib 400 mg, all taken once daily.	After imatinib treatment, the disease progressed. Gene mutation analysis revealed that the patient carried the E355G mutation, which is associated with imatinib resistance. The treatment was adjusted to nilotinib, and after 3 months, the patient achieved MCyR.	The E355G mutation leads to imatinib resistance in the patient but makes them sensitive to second-generation tyrosine kinase inhibitors, such as nilotinib.

ALT, alanine transaminase; CCyR, complete cytogenetic response; CHR, complete hematologic response; CMR, complete molecular response; ECOG, Eastern Cooperative Oncology Group; FFS, failure-free survival; EFS, event-free survival; MCyR, major cytogenetic response, MMR, major molecular response; MR4/DMR, log molecular response or deep molecular response; OS, overall survival; PFS, progression-free survival; Ph + CP-CML, philadelphia chromosome-positive chronic myeloid leukemia in chronic phase; qd, every day; 2G-TKI, second-generation tyrosine-kinase inhibitor.

Since this is the first detailed case report describing the use of flumatinib in treating CML patients with the rare e19a2 transcript, further research is required in larger sample cohorts to validate its efficacy and safety, addressing the limitations of single-case studies.

## Data Availability

The original contributions presented in the study are included in the article/supplementary material, further inquiries can be directed to the corresponding author.
